# Mental Health Treatment Rates During Pregnancy and Post Partum in US Military Service Members

**DOI:** 10.1001/jamanetworkopen.2024.13884

**Published:** 2024-05-30

**Authors:** Jennifer A. Heissel, Olivia J. Healy

**Affiliations:** 1 RAND Corporation, Santa Monica, California; 2 Department of Economics, Elon University, Elon, North Carolina

## Abstract

**Importance:**

Although new parents’ mental health is known to decline, less is known about changes in therapy attendance, especially among military service members.

**Objective:**

To investigate changes in therapy attendance among new parents and by parental leave length.

**Design, Setting, and Participants:**

This cohort study of US Army and Navy service members from January 1, 2013, to December 31, 2019, compared parents’ monthly therapy attendance with matched nonparents’ across childbirth and compared mothers’ weekly therapy attendance before vs after returning to work. Eligible monthly sample members included service members with first births from January 1, 2014, to December 31, 2017, and 12 months of data before to 24 months after birth and nonparents with 36 months of data. Eligible weekly sample members included mothers with first births from January 1, 2013, to June 30, 2019, and data from 12 months before to 6 months after birth and nonparents with 18 months of data. Data analysis was performed from July 1, 2023, to January 15, 2024.

**Exposure:**

Those exposed to parenthood had no prior children, acquired a dependent younger than 1 year, and, for mothers, had an inpatient birth. Unexposed matches did not add a dependent younger than 1 year.

**Main Outcomes and Measures:**

Monthly counts of mental health therapy sessions and any therapy sessions (weekly).

**Results:**

The monthly sample included 15 554 193 person-month observations, representing 321 200 parents and matches, including 10 193 mothers (3.2%; mean [SD] age, 25.0 [4.9] years), 50 865 nonmother matches (15.8%; mean [SD] age, 25.0 [5.0] years), 43 365 fathers (13.5%; mean [SD] age, 26.4 [4.8] years), and 216 777 nonfather matches (67.5%; mean [SD] age, 26.4 [4.8] years). The weekly sample included 17 464 mothers. Mothers went to 0.0712 fewer sessions at 1 month post partum (95% CI, −0.0846 to −0.0579) compared with 10 months before birth. Fathers went to 0.0154 fewer sessions in the month of birth (95% CI, −0.0194 to −0.0114) compared with 10 months before. Parents with preexisting treatment needs had larger decreases in treatment. Weekly therapy attendance increased by 0.555 percentage points (95% CI, 0.257-0.852) when mothers returned to work from 6 weeks of leave and 0.953 percentage points (95% CI, 0.610-1.297) after 12 weeks of leave.

**Conclusions and Relevance:**

In this cohort study of new parents, therapy attendance decreased around childbirth, especially among parents with prior mental health needs and mothers with longer maternity leaves. These findings suggest that more accessible treatment, including home visits or telehealth appointments, is needed.

## Key Points

**Question:**

How does US service members’ use of mental health treatment change across the transition to parenthood and return to work after parental leave?

**Findings:**

In this cohort study of 15 554 193 person-month observations, representing 321 200 parents and matches, US Army and Navy service members went to fewer mental health therapy sessions during pregnancy and immediately after a first birth, especially among those with a history of attending treatment. Mothers’ propensity to attend therapy remained lower while on parental leave and rebounded when they returned to work.

**Meaning:**

These findings suggest the transition to parenthood created barriers to mental health treatment and that return to work may have facilitated improved mental health treatment rates for mothers.

## Teaser

This cohort study of new parents in the US military investigates changes in use of mental health therapy sessions during pregnancy and post partum.

## Introduction

Pregnancy and the postpartum period are particularly risky times for parents’ mental health.^1,2^ Approximately 20% of mothers experience a depressive episode within 3 months post partum, with elevated risk beginning in pregnancy.^1,3,4,5^ Up to 20% of mothers experience anxiety during pregnancy, with continued risk post partum.^6,7,8,9^ Risk of severe mental health disorders, such as bipolar disorder, affective psychosis, and schizophrenia, is also elevated during pregnancy and post partum.^10^ Similarly, 8% to 10% of fathers had prenatal or postpartum depression, particularly 3 to 6 months post partum.^11,12^

Despite higher risk, parents’ use of mental health services is low.^13,14,15,16^ In a prospective study, only one-third of recent-birthing mothers with a diagnosable mental health disorder at their first prenatal appointment had contact with mental health services during pregnancy through 3 months post partum.^15^ Similarly, only one-third of mothers with major depressive disorder at their first prenatal appointment received any professional treatment by 6 weeks post partum.^14^ Research on fathers’ mental health care use is scarce but suggests limited use (eg, 3.2% of fathers in counseling among those with 3-year-old children).^16^

Barriers to mental health treatment occur at individual and structural levels.^17,18,19^ One reason for low use could be a lack of time to attend appointments. Childbirth increases time pressure on new parents, especially mothers.^20^ Pregnancy involves an increase in prenatal health care visits, which could crowd out time for other medical appointments. In theory, parental leave could alleviate time pressure, allowing parents to attend appointments. However, staying at home with an infant, especially in the early postpartum period, may mean alternative childcare for the infant is not available.

This cohort study investigated the use of mental health treatment during pregnancy and post partum among new parents in the US military. We focused on military service members for 3 reasons. First, this population faces increased risk of mental health difficulties and underuses mental health services absent parenthood.^21^ Second, the military provides no-cost health care coverage, meaning cost is not a barrier to treatment and mental health services may be more accessible across the transition to parenthood for service members compared with civilians. Third, the military maintains individual-level data on health care use and family formation and has expanded parental leave policies over time.^22^ These factors provided an opportunity to explore whether service members’ mental health care use changed when mothers returned to work after 6 vs 12 weeks of parental leave.

## Methods

### Study Design

This cohort study compared monthly changes in military parents’ use of mental health treatment with matched nonparents’ across childbirth separately for male and female service members. We also compared weekly propensity to attend therapy before vs after returning to work from leave for mothers. We followed the Strengthening the Reporting of Observational Studies in Epidemiology (STROBE) reporting guideline. The institutional review board at the Naval Postgraduate School approved this study and waived informed consent because the study was determined to be of minimal risk to participants.

### Study Population and Data

The eligible study population consisted of service members in the US Army and Navy who served on active duty sometime between January 1, 2013, and December 31, 2019. We obtained personnel data on service member demographics (age, sex, race and ethnicity, educational level, and Armed Forces Qualification Test scores, which measure aptitude and intelligence), job characteristics (rank, time in service, and branch of service), and dependent characteristics for spouses and children (date of birth, sex, and whether a spouse is in the military) through the Defense Enrollment Eligibility Reporting System (DEERS) database contained in the Army’s Person-Event Data Environment.^23^ Our indicator variables for identifying as Black or Hispanic were derived from the DEERS database, collected through self-report (eTable 1 in Supplement 1). Black and Hispanic were not mutually exclusive categories. “All other races” was used as the reference group for Black individuals given the difficulty of parsing multiple racial identities into mutually exclusive categories for analytic purposes; this group included American Indian or Alaska Native, Asian, Native Hawaiian or other Pacific Islander, White, and all combinations of these categories, including combinations with Black. We included measures of race and ethnicity in this study to account for differential patterns in mental health care treatment across groups. We connected DEERS data to Defense Health Agency records of all inpatient and outpatient clinical encounters in military or civilian treatment locations paid for by military health insurance (TRICARE); this dataset included billing codes, procedure codes, and encounter and admission dates.

Analyses relied on 2 samples. First, we used monthly data and required at least 12 months of continuous observations before birth and 24 months after for parents and 36 months of continuous observations for nonparents. When data were available for sample members 13 to 24 months before birth, we included those observations but did not require individuals to be observed in that time frame (eFigure 1 in Supplement 1). Second, we used weekly data, requiring at least 12 months of continuous observations before birth and 6 months after birth for parents and 18 weeks of continuous observations for nonmothers.

### Exposure

We defined exposure to the transition to parenthood as acquiring a new dependent younger than 1 year for those with no prior children and, for mothers, having had an inpatient birth-related hospital stay near the time of birth. The comparison group of nonexposed individuals (nonparents) had no children during (or before) the study window.^24,25^ We matched nonparents to parents by job rank, service branch, years of service, and other demographic characteristics, measured 10 months before birth (eAppendix 1 in Supplement 1). To avoid differences between parents and nonparents in loss to follow-up, our analyses focused on individuals who remained in the military for the full postbirth period of study.

### Outcomes

Our first outcome was the number of mental health therapy sessions per month among service members to study changes across the transition to parenthood. Our second outcome was an indicator for whether a service member went to any mental health therapy sessions in a given week (with 1 indicating yes and 0 indicating no) to study weekly changes before and after parental leave ended.^18^ We identified therapy sessions using *Current Procedural Terminology* billing codes for services provided by psychologists, psychiatrists, and other mental health professionals (eTable 2 in Supplement 1). The most common code was “Psychotherapy, 45 minutes.”

### Statistical Analysis

Data analysis was performed from July 1, 2023, to January 15, 2024. We estimated changes in mental health treatment across the transition to parenthood using a comparative interrupted times series approach in which pregnancy and birth each potentially interrupted time trends in the outcome. Nonparents were the unexposed comparison group, which allowed us to control for characteristics that parents and nonparents shared. We expected to observe no difference in outcomes between parents and nonparents before the pregnancy if matched nonparents were a suitable comparison group for parents.

Regression models estimated month-by-month differences between parents and nonparents in mental health treatment for 24 months before through 24 months after birth (eAppendix 1 in Supplement 1). Nonparents received a weight of 1/n, where n was the number of nonparents matched to a given parent (maximum n = 5). Models included heteroskedasticity-robust standard errors clustered by match group (each parent and their matches).^26^ Models were estimated separately for male and female service members using Stata software, version 15 (StataCorp LLC).^27^ Our minimum requirement for statistical significance was a 2-sided *P* < .05.

We also investigated whether changes in mental health care use were more pronounced for parents with prior mental health care needs, defined as having had 2 or more therapy sessions in the 10 to 24 months before birth. We restricted this analysis to a subsample of individuals who were continuously observed for 24 months before the birth (and 24 months after), allowing us to observe the total number of therapy sessions they attended 10 to 24 months before birth.

To estimate how mothers’ mental health treatment changed during and on return to work after parental leave, we studied week-by-week changes in the likelihood of going to therapy and whether there was a change (up or down) in the use of therapy the week when mothers returned to work. We estimated return-to-work changes separately for mothers under the 6-week vs 12-week leave policy and focused on trends in the 6 weeks surrounding the return to work (eAppendix 1 in Supplement 1). As an alternative approach, we reestimated models using linear time trends for all weeks 1 to 24 after the birth (eAppendix 2 in Supplement 1).

We focused on mothers with births from January 1, 2013, through June 30, 2019, excluding births to US Navy mothers from January 1, 2015, to December 31, 2016, when they may have qualified for an 18-week leave policy. Regression models estimated a 3-way interaction among linear time trends, an indicator for the time after leave (eg, weeks 7-12 for the 6-week policy), and an indicator for parents (1) vs comparisons (0). The model included a match-group fixed effect. We focused on the coefficient that measured the mother-specific change in therapy sessions on return to work (the post × parent interaction) (eAppendix 1 in Supplement 1). Models included heteroskedasticity-robust standard errors clustered by match group.^26^ The parental leave analysis only included female service members because fathers typically did not qualify for the 6- or 12-week parental leave policies. A final alternative estimate used mothers who had children during the same time in the calendar year but had different amounts of leave (6 vs 18 weeks) due to variation in policies across services branches (eAppendix 2 in Supplement 1).

## Results

The monthly sample included 15 554 193 person-month observations, representing 321 200 parents and matches, including 10 193 mothers (3.2%; mean [SD] age, 25.0 [4.9] years), 50 865 nonmother matches (15.8%; mean [SD] age, 25.0 [5.0] years), 43 365 fathers (13.5%; mean [SD] age, 26.4 [4.8] years), and 216 777 nonfather matches (67.5%; mean [SD] age, 26.4 [4.8] years). This sample included 61 258 Black parents or matches (19.1%), 259 942 of races other than Black (80.9%), 47 048 Hispanic parents or matches (14.6%), and 274 152 (85.4%) non-Hispanic. The weekly sample included 2 511 792 person-week observations of a sample of 104 658 comparisons, including 17 464 mothers (16.7%; mean [SD] age, 24.7 [4.7] years) and 87 194 nonmother matches (83.3%; mean [SD] age, 24.8 [4.8] years). This sample included 33 423 Black mothers or matches (31.9%), 71 235 of other races (68.1%), 18 235 Hispanic mothers or matches (17.4%), and 86 423 (82.6%) non-Hispanic. The weekly sample was larger than the monthly sample because fewer individuals were lost to follow-up data restrictions.

Differences between parents and matched nonparents across the 2 analytic samples were minimal (Table 1). Parents and nonparents had the same likelihood of being an officer and in the US Navy and the same average job rank by construction of our matching procedure. Mean months in service were very similar, given that we exact-matched on years in service. Parents and nonparents were also similar on other variables used to match parents with nonparents.

**Table 1.** Descriptive Baseline Statistics for Monthly and Weekly Analytic Samples for Parents and Matches^a^

Characteristic	Monthly sample^b^				Weekly sample, female service members^c^	
	Female service members		Male service members			
	Mothers (n = 10 193)^d^	Matched cohort (n = 50 865)^e^	Fathers (n = 43 365)^d^	Matched cohort (n = 216 777)^e^	Mothers (n = 17 464)^d^	Matched cohort (n = 87 194)^e^
Officer	1671 (16.4)	8339 (16.4)	7876 (18.2)	39 371 (18.2)	2649 (15.2)	13 226 (15.2)
US Navy	5475 (53.7)	27 321 (53.7)	20 902 (48.2)	104 487 (48.2)	7463 (42.7)	37 261 (42.7)
Time in service, mean (SD), mo	49.9 (45.8)	49.9 (45.9)	63.2 (48.3)	63.2 (48.3)	48.1 (42.8)	48.1 (42.9)
Age, mean (SD), y	25.0 (4.9)	25.0 (5.0)	26.4 (4.8)	26.4 (4.8)	24.7 (4.7)	24.8 (4.8)
Race						
Black	3317 (32.5)	16 227 (31.9)	7039 (16.2)	34 675 (16.0)	5661 (32.4)	27 762 (31.8)
All other races^f^	6876 (67.5)	34 638 (68.1)	36 326 (83.8)	182 102 (84.0)	11 803 (67.6)	59 432 (68.2)
Ethnicity						
Hispanic	1803 (17.7)	8970 (17.6)	5938 (13.7)	30 337 (14.0)	3027 (17.3)	15 208 (17.4)
Non-Hispanic	8390 (82.3)	41 895 (82.4)	37 427 (86.3)	186 440 (86.0)	14 437 (82.7)	71 986 (82.6)
Marital status						
Married	5503 (54.0)	26 782 (52.7)	31 942 (73.7)	158 488 (73.1)	9559 (54.7)	47 147 (54.1)
Military spouse	2610 (25.6)	11 985 (23.6)	2972 (6.9)	12 932 (6.0)	4663 (26.7)	21 756 (25.0)
Degree attainment						
Some college	958 (9.4)	4986 (9.8)	3593 (8.3)	18 537 (8.6)	1623 (9.3)	8334 (9.6)
College graduate	2341 (23.0)	11 503 (22.6)	10 167 (23.4)	50 809 (23.4)	3804 (21.8)	18 722 (21.5)
Missing educational data	18 (0.2)	72 (0.1)	132 (0.3)	636 (0.3)	30 (0.2)	106 (0.1)
Recent fitness *z* score, mean (SD)	0.0 (0.9)	0.0 (0.9)	0.2 (0.9)	0.2 (0.9)	0.0 (0.9)	0.0 (0.9)
AFQT^g^						
Mean (SD) score	48.0 (25.9)	48.2 (26.0)	52.0 (29.9)	52.6 (30.1)	56.5 (15.4)	56.4 (15.8)
Missing AFQT data	1639 (16.1)	8065 (15.9)	7944 (18.3)	39 366 (18.2)	2661 (15.2)	13 090 (15.0)

Abbreviation: AFQT, Armed Forces Qualification Test.

^a^ Data are presented as number (percentage) of participants unless otherwise indicated.

^b^ Monthly samples based on births from January 1, 2014, to December 31, 2017; sample observations required from at least 12 before to 24 months after birth.

^c^ Weekly samples based on births that qualified for 6 or 12 weeks of parental leave. This consists of births from January 1, 2014, through June 30, 2019, excluding births to US Navy mothers from January 1, 2015, to December 31, 2016, when they may have qualified for an 18-week leave policy.

^d^ Characteristics of parents 10 months before birth. Binary variables displayed as percentages. The SDs are given for nonbinary variables.

^e^ Baseline characteristics of matches at the time of the match (10 months before birth). All nonparent matches were exact matched on military grade (E1, E2, E3, E4, E5, E6, E7-E9, warrant officers, O1, O2, O3, O4-O9), branch (US Army or US Navy), and years of service. The propensity score–matching model included the listed characteristics plus 2-way interactions between these characteristics and the continuous variable for age and a binary variable equal to 1 for married.

^f^ All other races include American Indian or Alaska Native, Asian, Native Hawaiian or other Pacific Islander, White, and all combinations of these categories, including combinations with Black. See eTable 1 in Supplement 1 for additional details. Given the importance of acknowledging the multiplicity of racial and ethnic identities, Black and Hispanic were not coded as mutually exclusive groups. However, “all other races” was used as the reference group for Black individuals given the difficulty of parsing multiple racial identities into mutually exclusive categorical groups for analytic purposes.

^g^ The AFQT is a percentile score ranging from 1 to 100, with 1 indicating the first percentile and 100 indicating the 100th percentile.

**Figure 1.**
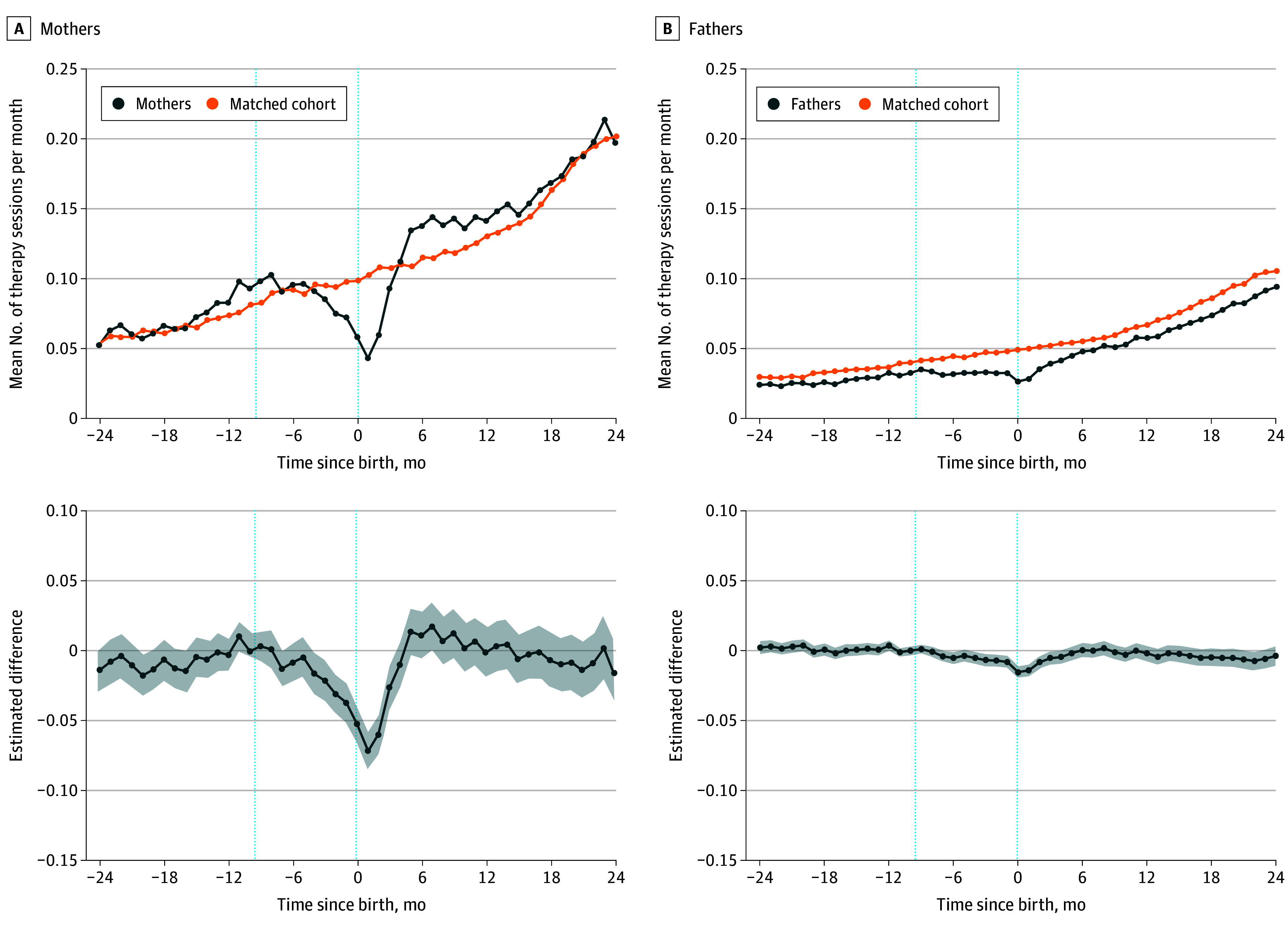
Changes in Mental Health Treatment Among Mothers and Fathers Across the Transition to Parenthood The outcome in the top row is the mean number of therapy sessions per month based on *Current Procedural Terminology* codes for health care services provided by a psychotherapist, psychiatrist, or other mental health professional. The bottom row shows the regression-adjusted estimate of the difference between the parents and matches in the outcome measured relative to 10 months before birth. Shaded areas indicate 95% CIs in the bottom row. Data include first births from January 1, 2014, to December 31, 2017; sample is required to be in the data from at least 12 months before to 24 months after birth. Dotted vertical lines at −9.5 and 0 indicate the start of the pregnancy period and the month of birth, respectively.

Unadjusted monthly trends in mental health treatment were similar between parents and nonparents until a few months before birth, when mothers and fathers began to attend fewer therapy sessions (Figure 1). In regression-adjusted estimates, the parent-nonparent difference in mental health treatment was statistically different from 0 starting 4 months before birth for mothers, with 0.0161 fewer sessions (95% CI, −0.0310 to −0.0012), and 7 months before the birth for fathers, with 0.0041 fewer sessions (95% CI, −0.0080 to −0.0003).

The decrease in therapy attendance was largest near the birth. At baseline, mothers and fathers went to 0.0929 and 0.0324 therapy sessions, respectively, 10 months before birth. Mothers went to 0.0712 fewer therapy sessions 1 month post partum (95% CI, −0.0846 to −0.0579) compared with 10 months before birth (−76.7%), after statistically accounting for time trends using the matches. The decrease for fathers was largest in the birth month, with 0.0154 fewer therapy sessions (95% CI, −0.0194 to −0.0114) compared with 10 months before (−47.5%).

By 4 months post partum, neither mothers’ nor fathers’ outcomes differed from their matches’ outcomes (−0.0097; 95% CI, −0.0255 to 0.0060 for mothers and −0.0047; 95% CI, −0.0095 to 0.00002 for fathers), signaling a return to typical rates of therapy attendance. No observations were lost to follow-up because we required the sample to remain in the military at least 2 years after birth (or the matched birth event).

**Figure 2.**
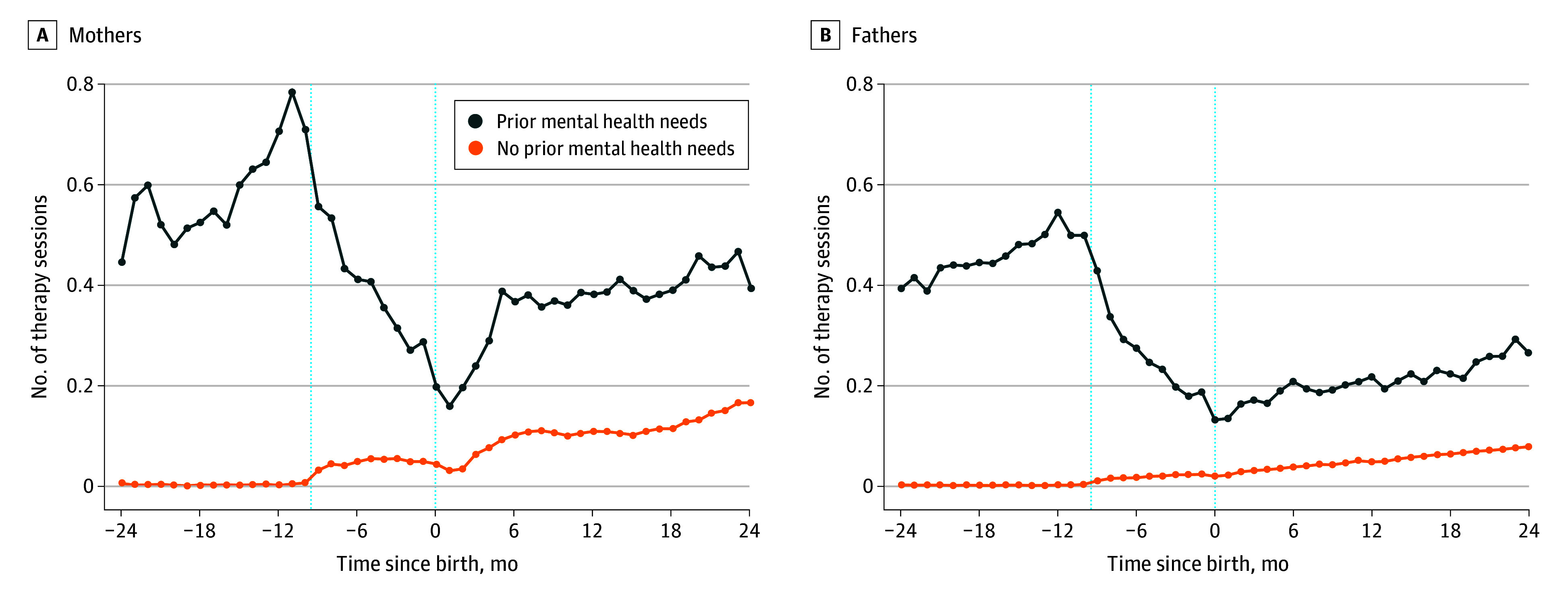
Changes in Mental Health Treatment Among New Mothers and Fathers by Prior Treatment History Data are the mean number of mental health therapy sessions per month among mothers and fathers who had at least 2 therapy sessions in the 10 to 24 months before birth (prior mental health needs) and mothers and fathers who had 0 or 1 sessions (no prior mental health needs). The x-axis is time in months relative to birth. Data include first births from January 1, 2015 (to allow observation of the prepregnancy data) to December 31, 2017; unlike in prior samples, this sample is required to be in the data from at least 24 months before to 24 months after birth. Dotted vertical lines at −9.5 and 0 indicate the start of the pregnancy period and the month of birth, respectively.

Decreases in mental health treatment were concentrated among parents with demonstrated prior need (Figure 2). Mothers with a history of at least 2 therapy sessions before pregnancy decreased from a mean (SD) of 0.7098 (1.4309) sessions 10 months before birth to 0.1601 (0.5772) sessions 1 month post partum (−77.4%). Fathers with a history of 2 or more therapy sessions decreased from a mean of 0.4994 (1.1031) sessions 10 months before birth to 0.1343 (0.6554) sessions 1 month post partum (−73.1%). Parents without at least 2 prior therapy sessions increased from 0.0074 (0.0857) sessions 10 months before birth to 0.0315 (0.2732) sessions 1 month after for mothers (+325.4%) and from 0.0043 (0.0655) sessions 10 months before birth to 0.0226 (0.2326) sessions 1 month after for fathers (+425.6%).

**Figure 3.**
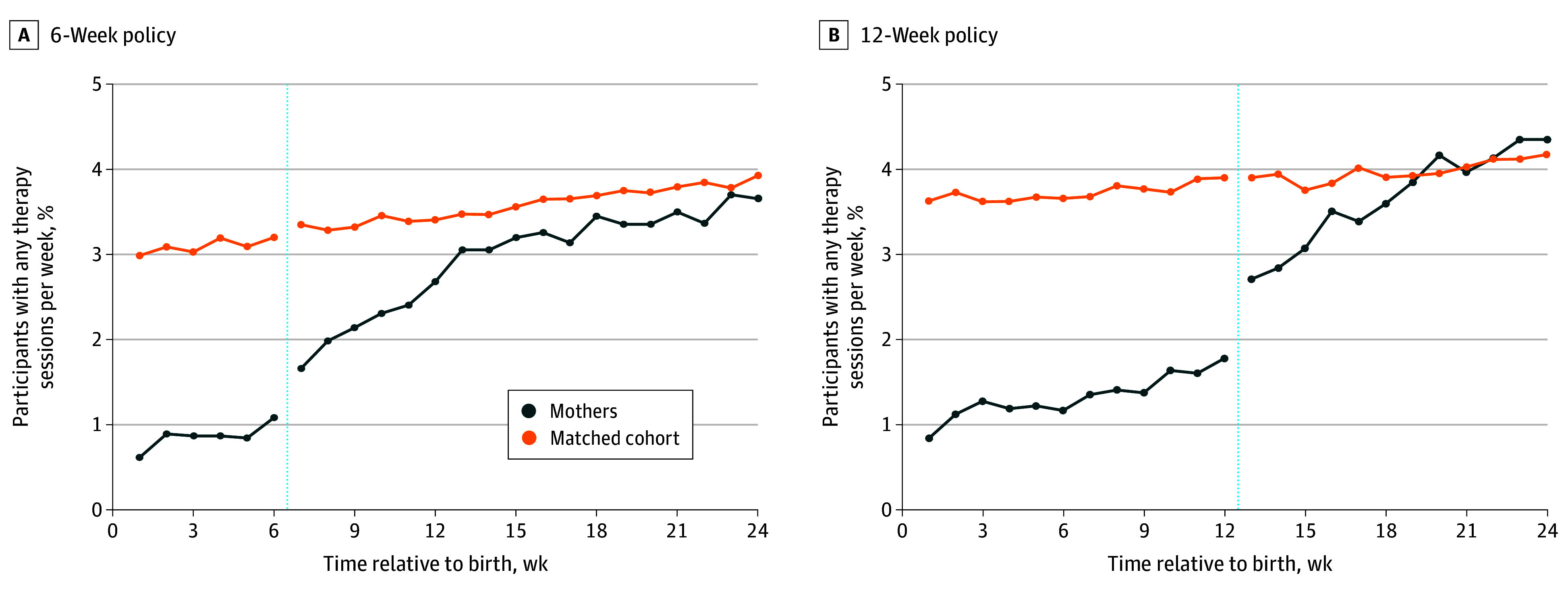
Mental Health Treatment Among US Army and Navy Mothers During 6- and 12-Week Parental Leave Policies Data are the mean percentage of mothers and their matches who attended at least 1 mental health therapy session in a given week relative to birth. Day 1 of week 1 begins the first full day after discharge from the hospital to match the start of parental leave tracking for administrative purposes. Dotted vertical lines indicate the end of parental leave and transition back to work.

Returning to work after parental leave was associated with increased therapy sessions (Figure 3). Under the 6-week policy, 1.08% of mothers attended a therapy session in week 6, the last week of leave. Regression-adjusted analyses (Table 2) showed this percentage increased by 0.56 percentage points (95% CI, 0.26-0.85 percentage points) at week 7 among mothers under the 6-week policy (51.3%) on mothers’ return to work. Under the 12-week policy, 1.78% of mothers attended a therapy session in week 12, the last week of leave. This increased by 0.95 percentage points (95% CI, 0.61-1.30 percentage points) at week 13 under the 12-week policy (53.4%) on mothers’ return to work (Table 2). The return-to-work change in therapy was larger in unadjusted models (Table 2) and when we fit time trends to all weeks of data before and after leave (eTable 3 in Supplement 1).

**Table 2.** Regression Results for the Return-to-Work Patterns for Mothers (Based on Diagnostic Codes)

Model	Outcome (95% CI)	Robust SE^a^	*P* value	No. of mothers^b^	Person-weeks^c^
**Return-to-work change, 6-wk policy**					
Unadjusted^d^	0.649 (0.396-0.902)	0.129	<.001	8322	99 864
Adjusted^e^	0.555 (0.257-0.852)	0.152	<.001	8322	598 476
**Return-to-work change, 12-wk policy**					
Unadjusted^d^	0.896 (0.591-1.200)	0.155	<.001	9142	109 704
Adjusted^e^	0.953 (0.610-1.297)	0.175	<.001	9142	657 420

^a^ Heteroskedasticity robust SEs clustered at the individual (unadjusted) or match-group (adjusted) level.

^b^ Data are the total number of unique mothers in the analysis (equivalent to the number of match groups).

^c^ Person-weeks is the total number of person-by-week observations included in the analysis for mothers (and matches in the adjusted analysis).

^d^ Unadjusted indicates no comparison to the match group or fixed effects. The outcome is an indicator of whether a person had a mental health therapy session in a given week. Coefficients shown are the estimated change at the end of leave (return to work) in an interaction between week since discharge from hospital and an indicator variable for post (ie, the end of leave).

^e^ Adjusted indicates a comparison to the match group and linear time trends. The outcome is an indicator of whether a person had a mental health therapy session in a given week. The analysis was adjusted for a 3-way interaction (and all lower-order terms) between week since discharge from hospital, an indicator variable for post (ie, after the end of leave), and an indicator variable for parent (equal to 1 for mothers and 0 for comparisons), as well as match-group fixed effects. Coefficients shown indicate the return-to-work change in the percentage of mothers going to therapy for mothers from the last week of leave to the first week back at work. Alternative models included in eAppendix 2 in Supplement 1.

We also compared therapy sessions between US Army mothers back at work after 6 weeks of leave and US Navy mothers in the same period who remained on leave through 18 weeks post partum. Unadjusted and regression-adjusted results were consistent with other return-to-work findings (eFigure 2 and eTable 4 in Supplement 1). US Army mothers back at work attended more therapy sessions than US Navy mothers, whose use of mental health treatment remained lower while on leave.

As an alternative outcome, we identified mental health care visits using medical billing diagnostic codes (eAppendix 3, eTable 5, and eTable 6 in Supplement 1). Results are similar for the monthly (eFigure 3 in Supplement 1) and weekly (eFigure 4 in Supplement 1) analyses.

## Discussion

Among service members in the US Army and Navy, parents’ use of mental health treatment decreased leading into a first birth and reached its lowest point around the time of birth. The general pattern of decrease was the same for mothers and fathers, but the size was larger for mothers. The frequency of therapy sessions recovered to that of matched comparisons by 4 months after birth for mothers and fathers. Decreases in mental health treatment around the transition to parenthood were concentrated among parents with a prior demonstrated need for services (ie, at least 2 therapy sessions before pregnancy).

Mental health treatment increased in the week mothers returned to work from parental leave. This increase in mental health treatment coincides with the start of childcare for mothers (needed for them to work) through military-subsidized or private arrangements (eg, a spouse, family members, or private market options). Many large bases have on-base subsidized childcare, so parents in this setting may be more able to put their child into formal childcare at the end of leave than civilians.

The postpartum period is known to be difficult for parents’ mental health,^1,3,4,5,6,7,8,9,10,11,12^ making it unlikely that the decreases we observed in therapy shortly before and after a first birth were due to better mental health. The finding that the decrease in therapy was largest among parents who had previously been attending therapy suggests lower treatment rates among a group with a demonstrated prior need for treatment.

Our findings are consistent with the idea that increased time constraints due to parenthood limit parents’ use of mental health services. Research has shown that the transition to parenthood is associated with increased time pressure, especially for mothers,^20^ suggesting that parents may have had less available time to go to therapy, even if needed. We found that mothers’ ability to attend therapy recovered once mothers returned to work from parental leave, further pointing to potential time constraints associated with being home on leave as a barrier to attending therapy sessions.

Prior literature interpreted decreases in mental health diagnoses in medical claims records during parental leave as an improvement in mental health.^28^ Our findings raise the alternative interpretation that caregiving responsibilities while on leave and lack of childcare create time constraints that limit parents’ ability to pursue care. Future research is needed to disentangle these 2 potential mechanisms.

A final alternate explanation for our observed findings might be that new financial constraints associated with parenthood make paying for mental health treatment more difficult for new parents, leading to less use. However, all service members in our study received military-funded, no-cost health insurance, meaning therapy sessions did not require any out-of-pocket costs. Therefore, decreases in therapy in our sample were unlikely due to the financial cost of mental health treatment when coupled with the added costs of child-rearing. Future research could explore whether financial barriers to mental health treatment exist for civilians around the transition to parenthood.

### Limitations

This study has several limitations. First, we examined mental health care use among active-duty US military service members, which may limit the generalizability of our results to civilians and individuals outside the US. Second, all service members had health insurance coverage and faced zero copayments for medical encounters. In turn, the findings may not generalize to parents who are uninsured or face copayments. Third, we could not fully disentangle whether decreases in therapy sessions reflected less need for treatment or an inability to access needed treatment, although subgroup analyses suggested that it was those who were likeliest to need care whose use decreased. Fourth, when studying changes at the end of leave, we observed only the length of leave mothers could have used, not whether they used all of it. Prior research has shown that military mothers during this period generally used all their available parental leave, but we cannot be sure for those in our sample.^22^ Fifth, we did not have random assignment of childbirth or parental leave policies. We used econometric strategies designed to produce plausible causal estimates of how parenthood and parental leave changed mental health treatment, but it is possible that our findings were spurious.

## Conclusions

Results from this cohort study indicate that the transition to parenthood decreased service members’ use of mental health treatment. Mothers and fathers attended fewer therapy sessions, starting a few months before and lasting a few months after the birth of their first child, possibly due to increased time demands associated with the transition to parenthood. Decreases in treatment were largest for those who attended therapy before the pregnancy, a group who may have most needed continued care. Mothers’ mental health treatment rates recovered after they returned to work from parental leave, indicating lack of childcare as a potential barrier to mental health treatment during leave. Findings suggest that both mothers and fathers could benefit from more accessible treatment, such as at-home mental health services (eg, home visits or telehealth appointments).^29^

## Notes

Supplement 1. **eTable 1.** Race and Ethnicity Codes Used to Code Black and Hispanic Variables **eAppendix 1.** Additional Details on Matching and Analyses **eFigure 1.** Analytic Sample Data Availability and Timeline Relative to Birth **eTable 2.** Procedure Codes Used to Identify Therapy Sessions for Mental Health Treatment **eAppendix 2.** Robustness Checks and Alternative Specifications for the Analysis of Changes After Returning to Work From Leave **eTable 3.** Alternative Models for the Return-to-Work Change in Propensity to Attend a Therapy Session After 6 and 12 Weeks of Parental Leave **eFigure 2**. Comparing Army Mothers (6 Weeks of Parental Leave) to Navy Mothers (18 Weeks of Parental Leave) **eTable 4.** Regression Estimates of the Return-to-Work Change in Therapy Sessions for Army Mothers After 6 Weeks of Leave Compared to Navy Mothers Still on Leave **eAppendix 3.** Alternative Outcome: ICD-9/ICD-10 Diagnostic Codes for Mental Health **eTable 5.** ICD-10 Diagnostic Codes Used to Identify an Alternative Mental Health Care Treatment Outcome **eTable 6.** ICD-9 Diagnostic Codes Used to Identify an Alternative Mental Health Care Treatment Outcome **eFigure 3.** Comparing Mothers and Fathers to Matches Across the Transition to Parenthood, Using Primary Diagnostic Codes to Define the Outcome **eFigure 4.** Comparing Army and Navy Mothers to Matches Across the End of the 6- and 12-Week Leave Policies, Using Primary Diagnostic Codes to Define the Outcome

Supplement 2.
Data Sharing Statement
