# Supplementary material for: Mental Health Treatment Rates During Pregnancy and Post Partum in US Military Service Members
Source: JAMA Netw Open. 2024 May 30;7(5):e2413884. doi: 10.1001/jamanetworkopen.2024.13884 (PMC11140539; doi:10.1001/jamanetworkopen.2024.13884)
Supplement: Supplement 1. — eTable 1. Race and Ethnicity Codes Used to Code Black and Hispanic Variables eAppendix 1. Additional Details on Matching and Analyses eFigure 1. Analytic Sample Data Availability and Timeline Relative to Birth eTable 2. Procedure Codes Used to Identify Therapy Sessions for Mental Health Treatment eAppendix 2. Robustness Checks and Alternative Specifications for the Analysis of Changes After Returning to Work From Leave eTable 3. Alternative Models for the Return-to-Work Change in Propensity to Attend a Therapy Session After 6 and 12 Weeks of Parental Leave eFigure 2. Comparing Army Mothers (6 Weeks of Parental Leave) to Navy Mothers (18 Weeks of Parental Leave) eTable 4. Regression Estimates of the Return-to-Work Change in Therapy Sessions for Army Mothers After 6 Weeks of Leave Compared to Navy Mothers Still on Leave eAppendix 3. Alternative Outcome: ICD-9/ICD-10 Diagnostic Codes for Mental Health eTable 5. ICD-10 Diagnostic Codes Used to Identify an Alternative Mental Health Care Treatment Outcome eTable 6. ICD-9 Diagnostic Codes Used to Identify an Alternative Mental Health Care Treatment Outcome eFigure 3. Comparing Mothers and Fathers to Matches Across the Transition to Parenthood, Using Primary Diagnostic Codes to Define the Outcome eFigure 4. Comparing Army and Navy Mothers to Matches Across the End of the 6- and 12-Week Leave Policies, Using Primary Diagnostic Codes to Define the Outcome [file jamanetwopen-e2413884-s001.pdf]

Supplementary Online Content

Heissel JA, Healy OJ. Mental health treatment rates during pregnancy and post partum in US military service members. *JAMA Netw Open*. 2024;7(5):e2413884. doi:10.1001/jamanetworkopen.2024.13884

eTable 1. Race and Ethnicity Codes Used to Code Black and Hispanic Variables

eAppendix 1. Additional Details on Matching and Analyses

eFigure 1. Analytic Sample Data Availability and Timeline Relative to Birth

eTable 2. Procedure Codes Used to Identify Therapy Sessions for Mental Health Treatment

eAppendix 2. Robustness Checks and Alternative Specifications for the Analysis of Changes After Returning to Work From Leave

eTable 3. Alternative Models for the Return-to-Work Change in Propensity to Attend a Therapy Session After 6 and 12 Weeks of Parental Leave

eFigure 2. Comparing Army Mothers (6 Weeks of Parental Leave) to Navy Mothers (18 Weeks of Parental Leave)

eTable 4. Regression Estimates of the Return-to-Work Change in Therapy Sessions for Army Mothers After 6 Weeks of Leave Compared to Navy Mothers Still on Leave

eAppendix 3. Alternative Outcome: ICD-9/ICD-10 Diagnostic Codes for Mental Health

eTable 5. ICD-10 Diagnostic Codes Used to Identify an Alternative Mental Health Care Treatment Outcome

eTable 6. ICD-9 Diagnostic Codes Used to Identify an Alternative Mental Health Care Treatment Outcome

eFigure 3. Comparing Mothers and Fathers to Matches Across the Transition to Parenthood, Using Primary Diagnostic Codes to Define the Outcome

eFigure 4. Comparing Army and Navy Mothers to Matches Across the End of the 6- and 12-Week Leave Policies, Using Primary Diagnostic Codes to Define the Outcome

This supplementary material has been provided by the authors to give readers additional information about their work.

© 2024 Heissel JA et al. *JAMA Network Open.*

eTable 1. Race and Ethnicity Codes Used to Code Black and Hispanic Variables

Black				Hispanic		
**Race Code**	**Definition**	**Value**		**Ethnic Code**	**Definition**	**Value**
001	American Indian/Alaska Native	0		AA	Asian Indian	0
002	Asian	0		AB	Chinese	0
003	Black or African American	1		AC	Filipino	0
004	Native Hawaiian or other Pacific Islander	0		AD	Guamanian	0
005	White	0		AF	Japanese	0
100	American Indian/Alaska Native, Asian	0		AG	Korean	0
101	American Indian/Alaska Native, Asian, Black or African American	0		AI	Vietnamese	0
102	American Indian/Alaska Native, Asian, Black or African American, Native Hawaiian or other Pacific Islander	0		AJ	Other Asian descent	0
103	American Indian/Alaska Native, Asian, Black or African American, Native Hawaiian or other Pacific Islander, White	0		AK	Mexican	1
104	American Indian/Alaska Native, Asian, Black or African American, White	0		AL	Puerto Rican	1
105	American Indian/Alaska Native, Asian, Native Hawaiian or other Pacific Islander	0		AM	Cuban	1
106	American Indian/Alaska Native, Asian, Native Hawaiian or other Pacific Islander, White	0		AN	Latin American with Hispanic descent	1
107	American Indian/Alaska Native, Asian, White	0		AO	Other Hispanic descent	1
108	American Indian/Alaska Native, Black or African American	0		AP	Aleut	0
109	American Indian/Alaska Native, Black or African American, Native Hawaiian or other Pacific Islander	0		AQ	Eskimo	0
110	American Indian/Alaska Native, Black or African American, Native Hawaiian or other Pacific Islander, White	0		AR	US or Canadian Indian tribes	0
111	American Indian/Alaska Native, Black or African American, White	0		AS	Melanesian	0
112	American Indian/Alaska Native, Native Hawaiian or other Pacific Islander	0		AT	Micronesian	0
113	American Indian/Alaska Native, Native Hawaiian or other Pacific Islander, White	0		AU	Polynesian	0
114	American Indian/Alaska Native, White	0		AV	Other Pacific island descent	0
115	Asian, Black or African American	0		BG	Other	0
116	Asian, Black or African American, Native Hawaiian or other Pacific Islander	0		BH	None	0
117	Asian, Black or African American, Native Hawaiian or other Pacific Islander, White	0		ZZ	Unknown	0
118	Asian, Black or African American, White	0				
119	Asian, Native Hawaiian or other Pacific Islander	0				
120	Asian, Native Hawaiian or other Pacific Islander, White	0				
121	Asian, White	0				

© 2024 Heissel JA et al. *JAMA Network Open.*

122	Black or African American, Native Hawaiian or other Pacific Islander	0				
123	Black or African American, Native Hawaiian or other Pacific Islander, White	0				
124	Black or African American, White	0				
125	Native Hawaiian or other Pacific Islander, White	0				
999	Unknown	0				

Notes: Displays the racial/ethnic categories in the DEERS data, based on self-reports to the military. Value indicates the value the Black and Hispanic variables were given in this analysis based on the DEERS data. Given the importance of acknowledging the multiplicity of racial and ethnic identities, Black and Hispanic were not coded as mutually exclusive categories. However, "all other races" was used as the reference group for Black individuals given the difficulty of parsing multiple racial identities into mutually exclusive categorical groups for analytic purposes.

© 2024 Heissel JA et al. *JAMA Network Open.*

eAppendix 1. ADDITIONAL DETAILS ON MATCHING AND ANALYSES

Matching Parents to Observably Similar Nonparents

Our matching strategy proceeded in three steps. First, we matched each parent to all the same-sex nonparents who had the same number of years of service, the same job rank, in the same service branch (Army or Navy) in the same month and year 10 months before the parent had their first child. For example, for a father in the Navy whose first child was born in March of 2015, who had 4 years of service and held the rank of Petty Officer Third Class (E4) in May 2014, we would isolate all men in the Navy *without* births during our study window who also had 4 years of service and the rank of Petty Officer Third Class (E4) in May of 2014 as potential matches. In our study setting, years of service, rank, and branch determined the day-to-day work environment, so we wanted to limit our comparisons to those who, for example, were likely to have similar freedom to go to a medical appointment.

Second, we further restricted potential nonparent matches within a month-year/branch/rank/years of service cell based on whether they were observably similar to the first-time parents on other dimensions. To do this, we used a logit model to estimate probability of having first birth in our sample, separately for male and female servicemembers. Key variables in our logit model included age; education (college degree, some college, and missing education relative to high school degree); most recent physical performance score measured in standard deviation units by branch, year, and sex; AFQT scores[*]; indicators for officer (relative to non-officers), Navy (relative to Army); time in service (measured in months); Black (relative to non-Black); Hispanic (relative to non-Hispanic); married (relative to unmarried); whether a spouse was also in the military; and interactions with all listed variables by age and marital status. The variables related to race and ethnicity were derived from the DEERS database, collected through self-report as described in eTable 1. These questions were queried separately and are not mutually exclusive categories. We included variables for Black and Hispanic because these variables may relate to use of mental health counselling services, and we wanted the parent and comparisons to be comparable on this measure.

We measured all characteristics for first-time parents 10 months before the observed first birth (i.e., prepregnancy). We then used this logit model to estimate a propensity score for each parent and nonparent. Among cells of parents and nonparents in the same month-year, branch, job rank, and number of years of service, we used nearest neighbor matching with replacement to select up to five nonparents for each parent that were the closest match in terms of their estimated propensity to have a baby 10 months later. This strategy meant the same nonparent could be matched to different first-time parents, or parents could match to less than 5 nonparents, who then got higher weights in the analysis.

Our third step in the matching process was to assign matched nonparents to a “placebo” birth event 10 months after the match. Our analyses could then compare the changes in outcomes for first-time parents to the average change for the matched nonparents. Each parent received a weight of 1 in the analysis, while each nonparent received a weight w of w=1/n, where n was the number of distinct nonparents in the match set.

[*] We had missing AFQT score data for many officers, who generally took GCT exams, an alternate test option. We assigned those with missing AFQT scores the mean value of AFQT and also included an indicator variable for missing AFQT.

© 2024 Heissel JA et al. *JAMA Network Open.*

Statistical Models – Monthly Changes in Outcomes Across Parenthood

For the month-level analysis, we created a series of datasets across exact-match groups (defined by the month-year, branch, job rank, and year of service at the time of the match). We required all sample members to be continuously observed between 12 months before through 24 months after the birth/placebo birth. We included any available data on sample members between 24 to 13 months before the birth, but for individuals who were not yet in the military or who gave birth early in the study window we did not always have this prepregnancy window of data. See eFigure 1 for a visual summary. Using all available month-year timepoints from 24 months before through 24 months after the birth/placebo birth, we then stacked exact-match-group datasets.[†]

eFigure 1. Analytic Sample Data Availability and Timeline Relative to Birth

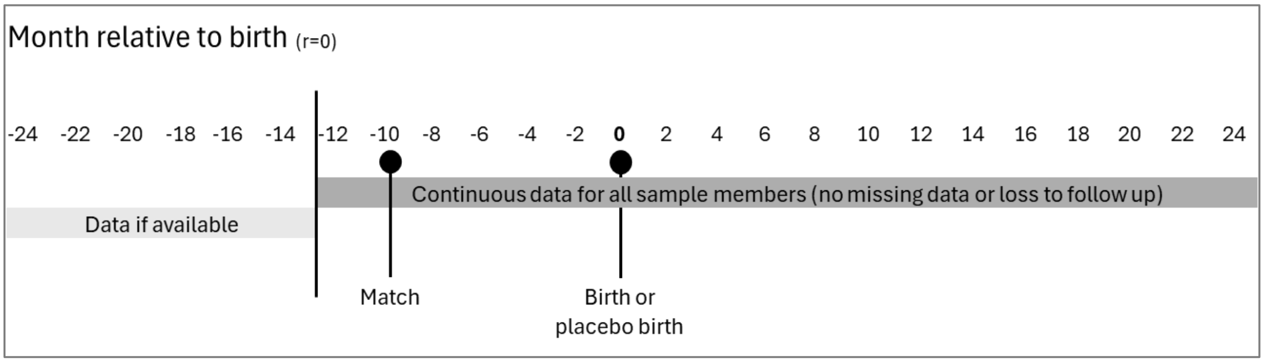

We estimated a fully flexible comparative interrupted time series specification (also known as an "event-study" specification) as follows:

$$Y_{igr} = \pi P_i + \sum_{r=-24}^{24} \mathbf{1}[(t - t_{ig}^{*} + r)P_i]\,\beta_r + \alpha_g + \tau_t + \varepsilon_{igr} \qquad \text{Eq. 1}$$

where t_{ig}^{*} was the month-year of the real (or placebo) birth for individual *i* in match group *g* based on calendar time *t*. We measured the month relative to birth as r. P_i was equal to one for all first-time parents and zero for matched nonparents; we expected π to equal zero given that parents and matched nonparents were similar in the preperiod. Calendar month-year fixed effects τ_t accounted for any time-varying trends; because we matched parents to nonparents in the same month and year these fixed effects were synonymous with month-relative-to-match effects. We included α_g to create a within-match-group comparison and ε_{igr} as the error term. Then, β_r represented how much the parents differed from their matches at a particular time *r*. Coefficients were measured relative to *r* = -10, which represented 10 months before birth and approximately 1 month before the start of the pregnancy. We focused on month-by-month patterns starting 24 months before through 24 months after birth, dropping *r* < -24 and *r* > 24. Then, for example, β_{12} estimated whether parents' outcomes were larger, smaller, or the same as the nonparent matches in their match group at 12 months after the birth (22 months after the match).

[†] Each month-year observed for a given parent appeared exactly once as they had only one first birth. A given month-year for nonparents may have occurred multiple times if the same non-nonparent was matched in different cell to a different parent. This matched individual would have had different relative time points in the analysis as defined by the time point of their match and assigned placebo birth.

Eq. 1 allowed us to estimate prepregnancy differences in outcomes between first-time parents and nonparents, reflected by β_r estimates when *r*<-10. Our matching procedure did not mechanically require outcomes between parents and their matches to move together when *r*<-10, but evidence of parallel trends in outcomes β_r for *r*=[-24, -11] increased confidence that the matched comparison group was a reasonable approximation of outcomes for parents.

We clustered standard errors at the match-group level, given prior research that clustering should be at the match-group level to account for within-group correlation induced by the matching procedure.[26] Given the sample size and computational limitations of the remote servers we used to analyze this data, we could not conduct two-way clustering at the match-group and individual levels.

Statistical Models – Weekly Changes in Outcomes After the Return to Work from Parental Leave

Our unadjusted model to estimate weekly changes in outcomes at the end of parental leave was:

$$Y_{iw} = \pi + \beta \cdot f(weeks_{iw}) + \delta \cdot \mathbf{1}(weeks_{iw} > leaveend_i) + \varepsilon_{iw} \,,$$
$$with\ |weeks_{iw} - leaveend_i| \leq k \qquad \text{Eq. 2}$$

where, for individual *i*, the outcome Y_{iw} was an indicator for whether an individual obtained any mental health treatment in the week relative to birth *w*. The variable $weeks_{iw}$ was measured relative to the time since the individual was discharged from the hospital (with *w*=1 starting on the first full day at home following hospital discharge). Then, $f(weeks_{iw})$ was a linear function of the $weeks_{iw}$variable, which we allowed to differ on either side of the end of leave. The variable $leaveend_i$ was defined as the applicable number of weeks of leave available (6 or 12) based on the date of birth and service branch and $\mathbf{1}(weeks_{iw} > leaveend_i)$ was an indicator for the weeks after the end of leave. The value k defined the window of data (e.g., w = [1, 24] or w = [7, 18]) used in the analysis. Our primary specification set *k=6*, meaning we used 6 weeks of data on either side of the end of leave to estimate the model. The coefficient of interest is δ, which provides an estimate of how much health care treatment changes from the last week of leave to first week the mother returns to work.

Our model for weekly changes in outcomes at the end of parental leave that adjusts for confounders adds in a group of comparable nonparents. This model estimates the following:

$$Y_{igw} = \pi + \beta \cdot f\left(weeks_{igw}\right) + \delta \cdot \mathbf{1}\left(weeks_{igw} > leaveend_g\right) + \varphi \cdot P_i$$
$$+\gamma \cdot \left[\mathbf{1}\left(weeks_{igw} > leaveend_g\right) \cdot (P_i)\right] + \alpha_g + \varepsilon_{iGw},$$
$$with\ |weeks_{iw} - leaveend_i| \leq k \qquad \text{Eq. 3}$$

where, for individual *i* in matched group g, the outcome Y_{igw} was an indicator for whether an individual obtained healthcare treatment in a given week relative to birth *w*; the variable $weeks_{igw}$ was measured relative to the time since the mother in the matched group was discharged from the hospital; $f\left(weeks_{igw}\right)$ was a linear function of the $weeks_{igw}$ variable, which we allowed to differ on either side of the end of leave and for mothers and matches; $leaveend_g$ was defined by the applicable number of weeks of leave available (6 or 12) based on the date of birth and service branch of the mother in the matched group; $\mathbf{1}\left(weeks_{igw} > leaveend_g\right)$ was an indicator for being after the end of leave; P_i was an

© 2024 Heissel JA et al. *JAMA Network Open.*

indicator equal to one if the person was a mother (and 0 otherwise); and α_g was a matched group fixed effect to account for group-specific, time-invariant characteristics (both observed and unobserved) common to each matched group. The coefficient of interest was γ, which provided an estimate of how much health care treatment changed between the last week of leave and the first week the mother returned to work, after accounting for general changes experienced by other members of the matched group (δ).

© 2024 Heissel JA et al. *JAMA Network Open.*

eTable 2. Procedure Codes Used to Identify Therapy Sessions for Mental Health Treatment

CPT Code	Description
90785	Psychotherapy complex interactive
90791	Psychiatric Diagnostic Evaluation
90792	Psychiatric Diagnostic Evaluation with medical services
90832	Psychotherapy, 30 minutes (16-37 minutes)
90833	Psychotherapy with evaluation and management, 30 minutes (16-37 minutes)
90834	Psychotherapy, 45 minutes (38-52 minutes)
90836	Psychotherapy with evaluation and management, 45 minutes (38-52 minutes)
90837	Psychotherapy, 60 minutes (53 minutes and over)
90838	Psychotherapy with evaluation and management 60 minutes (53 minutes and over)
90839	Psychotherapy for a crisis, 60 minutes (30-74 minutes)
90840	Psychotherapy for crisis; each additional 30 minutes
90845	Psychoanalysis
90846	Family or couples psychotherapy, without the patient present
90847	Family or couples psychotherapy, with the patient present
90853	Group Psychotherapy (not family)

© 2024 Heissel JA et al. *JAMA Network Open.*

eAppendix 2. ROBUSTNESS CHECKS AND ALTERNATIVE SPECIFICATIONS FOR THE ANALYSIS OF CHANGES AFTER RETURNING TO WORK FROM LEAVE

We re-estimated the jump in weekly leave use upon return to work by fitting time trends to all weeks of data before and after leave in the 1 to 24 weeks postpartum (eTable 2). We present unadjusted results (column 1), adjusted results (column 2), and the main specification from the article (column 3). With the alternative model that uses 1 to 24 weeks postpartum in the estimation, trends in the outcome at more distant time points (e.g., weeks farther away from the end of leave) influence the estimate. The size of the return-to-work jump was larger with this alternative specification.

eTable 3. Alternative models for the return-to-work change in propensity to attend a therapy session after 6 and 12 weeks of parental leave

Model	Model 1[b]	Model 2[c]	Model 3[d] (Primary in article)
# of weeks around leave end	All	All	+/-6 weeks
Includes comparisons	No	Yes	Yes
**Panel A: 6-week policy**			
Return-to-work jump (week 7)[a]	0.964***	0.907***	0.555***
	[0.710–1.218]	[0.618–1.197]	[0.257–0.852]
Mothers[e]	8,322	8,322	8,322
Person-weeks[f]	199,728	1,196,952	598,476
R-squared	0.2548	0.0816	0.0809
Mean, mothers at week 6	1.081	1.081	1.081
**Panel B: 12-week policy**			
Return-to-work jump (week 13)[a]	1.055***	1.104***	0.953***
	[0.796–1.314]	[0.820–1.388]	[0.610–1.297]
Mothers[e]	8,322	8,322	8,322
Person-weeks[f]	199,728	1,196,952	598,476
R-squared	0.2413	0.0774	0.0934
Mean, mothers at week 12	1.783	1.783	1.783

*** p < 0.001, **p < 0.01, *p < 0.05

[a] The outcome is an indicator for whether a person went to any mental health therapy sessions in a given week. The coefficient shown indicates the return-to-work jump in sessions for mothers from the last week of leave to the first week back at work.
[b] Model includes a two-way interaction (and all lower-order terms) between week since hospital discharge and an indicator variable for post (i.e., the end of leave), as well as individual fixed effects. Includes all 24 weeks following hospital discharge and does not include comparison group of non-mothers. Heteroskedasticity robust standard errors clustered at the individual level.
[c] Model includes a three-way interaction (and all lower-order terms) between week since hospital discharge, an indicator variable for post (i.e., the end of leave), and an indicator variable for parent (equal to 1 for mothers and 0 for comparisons), as well as match-group fixed effects. Includes all 24 weeks following the release from the hospital and includes comparison group of non-mothers. Heteroskedasticity robust standard errors clustered at the match-group level.
[d] Model includes a three-way interaction (and all lower-order terms) between week since hospital discharge, an indicator variable for post (i.e., the end of leave), and an indicator variable for parent (equal to 1 for mothers and 0 for comparisons), as well as match-group fixed effects. Includes +/-6 weeks from the end of leave and includes comparison group of non-mothers. Heteroskedasticity robust standard errors clustered at the match-group level.
[e] Mothers indicates the total unique mothers in the analysis; it is equivalent to the number of match groups.
[f] Person-weeks is the total number of person by week observations included in the analysis for mothers and matches.

Alternative Comparison Group and Estimation Procedure for Return-to-Work Analysis

We also used an alternative analytic strategy to estimate changes in mental health treatment beyond the acute jump at the return-to-work time point. This analysis used mothers who had babies in the military during the same time period but who had different amounts of leave due to variation in policies across services branches. Specifically, in early July 2015, the Department of Defense announced Navy birthing mothers would receive 18 weeks of leave (retroactive to babies born as of January 1, 2015). Army mothers were not eligible for the expansion and continued to only have 6 weeks until December 22, 2015 when the policy again changed. As a result, during this timeframe mothers in the Army had 6 weeks of leave and returned to work thereafter, while mothers in the Navy had 18 weeks of leave before returning to work. Using this variation, we compared differences in mental health treatment between Army mothers and Navy mothers during the stretch of weeks postpartum where Navy mothers remained on leave and Army mothers returned to work. The model used Navy mothers' outcomes (while they continued on extended parental leave) to approximate what would have happened to Army mothers' outcomes had they not gone back to work, net of any general Army-Navy differences.

We analyzed the average difference in outcomes in two ways: (1) for the 6 weeks after Army mothers returned to work (postpartum weeks 7 to 12), and (2) for the entire 12 weeks during which Army mothers were at work and Navy mothers continued on leave (postpartum weeks 7 to 18).

We estimated the following statistical model:

$$Y_{iw} = \pi \cdot (offleave_i) + \rho_i + \tau_w + \varepsilon_{iw} \qquad \text{Eq. 4}$$

where ρ_i controlled for characteristics that were constant within mothers and τ_w controlled for characteristics that were constant within weeks of time. The parameter π on the *offleave* variable was an estimate of the mean difference in outcomes between Navy mothers and Army mothers during the entire stretch of time where Army mothers were back and work and Navy mothers remained on leave. If Army mothers had more weeks with mental health treatment during the period they were back at work, beyond general differences in the Army's and Navy's use of mental health treatment (which the model controlled for), then we expected the coefficient π on the *offleave* variable would be positive.

The analysis focused on mothers with births from May 21, 2015, and December 21, 2015. Analyses excluded mothers who gave birth in January 1, 2015 – May 20, 2015 and their matches because some of these mothers (if in the Navy) received extra parental leave after they had already returned to work.[22]

Below, we show a visual of the trends in the unadjusted average share of mothers attending at least one therapy session each week, split by Army mothers with 6 weeks of leave and Navy mothers with 18 weeks of leave (Figure e1). The regression-adjusted results follow (Table e3) and present the average percentage difference in the odds of attending a therapy session across weeks 7-12 (Column 1) or weeks 7-18 (Column 2). Because this strategy did not focus on the acute jump after leave ended, the results were less affected by the value of the outcome in any given week after leave ended. On average, Army mothers whose parental leave ended after week 6 were 1.319 percentage points more likely to attend a therapy session in weeks 7-12 (95% CI: 0.711, 1.927); this represented an 184% increase over Navy mothers' average sessions in weeks 7-12. When we examined the full range of data from weeks 1-24, Army mothers were 0.655 percentage points more likely to attend a therapy session in weeks 7-18 (95% CI: 0.121, 1.188), covering the entire stretch when Army mothers were back at work while Navy mothers remained on leave. This increase represented a 77% increase over Navy mothers' average in weeks 7-18.

© 2024 Heissel JA et al. *JAMA Network Open.*

eFigure 2. Comparing Army Mothers (6 weeks of parental leave) to Navy Mothers (18 weeks of parental leave)

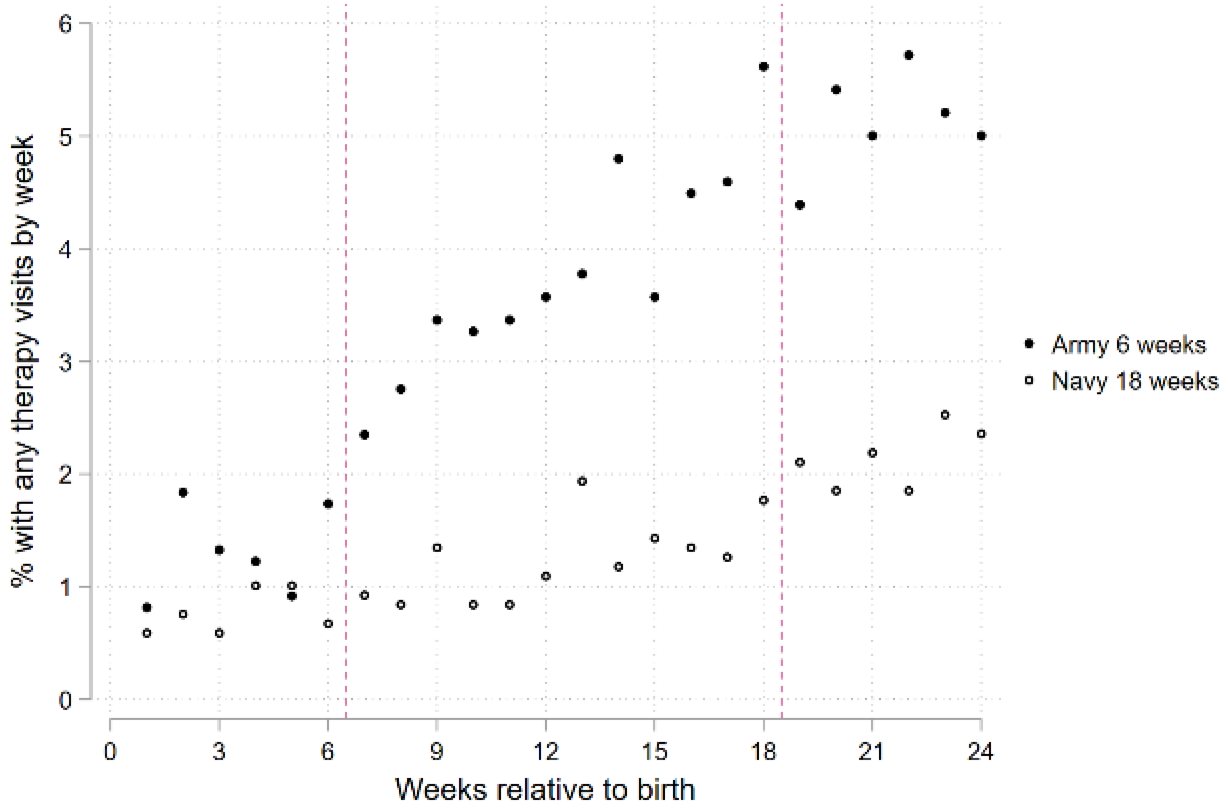

Notes: Displays the mean percent of mothers who had at least one mental health therapy session by week relative to birth, by Army and Navy mothers for first births between May 21, 2015 and December 21, 2015. Army birth mothers were allowed 6 weeks of leave during this time; Navy mothers were allowed 18 weeks. Day 1 of week 1 begins the first full day after discharge from the hospital to match the start of parental leave tracking for administrative purposes. Navy mothers did not have to use weeks 7-18 of leave continuously; they could use it anytime before the child's first birthday.

© 2024 Heissel JA et al. *JAMA Network Open.*

eTable 4. Regression estimates of the return-to-work change in therapy sessions for Army mothers after 6 weeks of leave compared to Navy mothers still on leave

Model	Model 1[a]	Model 2[b]
Off leave (avg., weeks 7-12)	1.594***	
	[0.879–2.310]	
Off leave (avg., weeks 7-18)		0.803**
		[0.203–1.404]
Mothers[c]	2,167	2,167
Person-weeks[d]	26,004	52,008
R-squared	0.2864	0.2430
Mean, Navy mothers weeks 7-12	0.982	
Mean, Navy mothers weeks 7-18		1.235

*** p < 0.001, **p < 0.01, *p < 0.05

[a] The outcome is an indicator for whether a person attended a mental health therapy session in a given week. Coefficient shown indicates the mean difference between Army and Navy mothers during the 7-12 weeks when the Navy remained on leave and the Army had returned to work. Model includes individual and week fixed effects. Includes first 12 weeks following hospital discharge and does not include comparison group of non-mothers. Heteroskedasticity robust standard errors clustered at the individual level.

[b] The outcome is an indicator for whether a person attended a mental health therapy session in a given week. Coefficient shown indicates the mean difference between Army and Navy mothers during the 7-12 weeks when the Navy remained on leave and the Army had returned to work. Model includes individual and week fixed effects. Includes first 24 weeks following hospital discharge and does not include comparison group of non-mothers. Heteroskedasticity robust standard errors clustered at the individual level.

[c] Mothers indicates the total unique mothers in the analysis; it is equivalent to the number of match groups.

[d] Person-weeks is the total number of person-by-week observations included in the analysis for mothers and matches.

eAppendix 3. ALTERNATIVE OUTCOME: ICD-9/ICD-10 DIAGNOSTIC CODES FOR MENTAL HEALTH

As an alternative strategy to identify receipt of mental health-related medical care, we used billing codes from the *International Statistical Classification of Diseases and Related Health Problems, Ninth Revision and Tenth Revisions* (ICD-9 and ICD-10). We relied on codes from the ICD-10 "Mental and Behavioral Disorders" chapter and ICD-9 codes under the Major Diagnostic Category (MDC) 19 on "Mental Diseases and Disorders." The specific codes used (included any excluded) are presented in eTable 5 and 6 below. When using diagnosis codes to define mental health care use, we focused on medical encounters where the admission reason (inpatient) or first diagnostic code (outpatient) was a mental health-related code, meaning mental health was the primary concern at the visit. We re-estimated the month-by-month analysis for male and female servicemembers across the transition to parenthood (eFigure 3) and present the descriptive trends for the week-by-week analysis for female servicemembers upon return-to-work from leave (eFigure 4). Like the primary results, parents and especially mothers obtained less mental health-related care under the alternative definition of the outcome and mothers saw a jump in use of mental health care upon the return to work from leave.

eTable 5. ICD-10 Diagnostic Codes Used to Identify an Alternative Mental Health Care Treatment Outcome

ICD10 Mental Health Diagnosis Codes Included		Excluded ICD10 Diagnosis Codes
F00–F09	Organic, including symptomatic, mental disorders	F0781 (Postconcussional syndrome); F0789 (Other personality and behavioral disorders due to known physiological condition)
F10–F19	Mental and behavioral disorders due to psychoactive substance abuse	
F20–F29	Schizophrenia, schizotypal, and delusional disorders	
F30–F39	Mood disorders, depression, and bipolar disorders	
F40–F49	Neurotic, anxiety, stress-related, and somatoform disorders	F482 (Pseudobulbar affect)
F50–F59	Behavioral syndromes associated with physiological disturbances and physical factors	F525 (Vaginismus not due to a substance or known physiological condition); F550 (Abuse of antacids); F554 (Abuse of vitamins)
F60–F69	Disorders of adult personality and behaviors	
F70–F79	Intellectual disabilities	
F80–F89	Pervasive and specific developmental disorders	F8081 (Childhood onset fluency disorder); F842 (Rett's syndrome); F843 (Other childhood disintegrative disorder)
F90–F98	Behavioral and emotional disorders with onset usually occurring in childhood and adolescence	F951 (Chronic motor or vocal tic disorder); F952 (Tourette's disorder); F958 (Other tic disorders); F959 (Tic disorder, unspecified)
F99	Unspecified mental disorder	

© 2024 Heissel JA et al. *JAMA Network Open.*

eTable 6. ICD-9 Diagnostic Codes Used to Identify an Alternative Mental Health Care Treatment Outcome

ICD9 Mental Health Diagnosis Codes Included	
290-294	Organic Psychotic Conditions
295-299	Other Psychoses
300-316	Neurotic Disorders, Personality Disorders, And Other Nonpsychotic Mental Disorders
317-319	Intellectual Disabilities

Excluded ICD9 Codes	Description of Excluded Codes
2910	Alcohol withdrawal delirium
2911	Alcohol-induced persisting amnestic disorder
2912	Alcohol-induced persisting dementia
2913	Alcohol-induced psychotic disorder with hallucinations
2914	Idiosyncratic alcohol intoxication
2915	Alcohol-induced psychotic disorder with delusions
29181	Alcohol withdrawal
29182	Alcohol induced sleep disorders
29189	Other alcohol-induced mental disorders
2919	Unspecified alcohol-induced mental disorders
2920	Drug withdrawal
29211	Drug-induced psychotic disorder with delusions
29212	Drug-induced psychotic disorder with hallucinations
2922	Pathological drug intoxication
29281	Drug-induced delirium
29282	Drug-induced persisting dementia
29283	Drug-induced persisting amnestic disorder
29284	Drug-induced mood disorder
29285	Drug induced sleep disorders
29289	Other specified drug-induced mental disorders
2929	Unspecified drug-induced mental disorder
30300	Acute alcoholic intoxication in alcoholism, unspecified
30301	Acute alcoholic intoxication in alcoholism, continuous
30302	Acute alcoholic intoxication in alcoholism, episodic
30303	Acute alcoholic intoxication in alcoholism, in remission
30390	Other and unspecified alcohol dependence, unspecified
30391	Other and unspecified alcohol dependence, continuous
30392	Other and unspecified alcohol dependence, episodic
30393	Other and unspecified alcohol dependence, in remission
30400	Opioid type dependence, unspecified
30401	Opioid type dependence, continuous
30402	Opioid type dependence, episodic

© 2024 Heissel JA et al. *JAMA Network Open.*

Excluded ICD9 Codes	Description of Excluded Codes
30403	Opioid type dependence, in remission
30410	Sedative, hypnotic or anxiolytic dependence, unspecified
30411	Sedative, hypnotic or anxiolytic dependence, continuous
30412	Sedative, hypnotic or anxiolytic dependence, episodic
30413	Sedative, hypnotic or anxiolytic dependence, in remission
30420	Cocaine dependence, unspecified
30421	Cocaine dependence, continuous
30422	Cocaine dependence, episodic
30423	Cocaine dependence, in remission
30430	Cannabis dependence, unspecified
30431	Cannabis dependence, continuous
30432	Cannabis dependence, episodic
30433	Cannabis dependence, in remission
30440	Amphetamine and other psychostimulant dependence, unspecified
30441	Amphetamine and other psychostimulant dependence, continuous
30442	Amphetamine and other psychostimulant dependence, episodic
30443	Amphetamine and other psychostimulant dependence, in remission
30450	Hallucinogen dependence, unspecified
30451	Hallucinogen dependence, continuous
30452	Hallucinogen dependence, episodic
30453	Hallucinogen dependence, in remission
30460	Other specified drug dependence, unspecified
30461	Other specified drug dependence, continuous
30462	Other specified drug dependence, episodic
30463	Other specified drug dependence, in remission
30470	Combinations of opioid type drug with any other drug dependence, unspecified
30471	Combinations of opioid type drug with any other drug dependence, continuous
30472	Combinations of opioid type drug with any other drug dependence, episodic
30473	Combinations of opioid type drug with any other drug dependence, in remission
30480	Combinations of drug dependence excluding opioid type drug, unspecified
30481	Combinations of drug dependence excluding opioid type drug, continuous
30482	Combinations of drug dependence excluding opioid type drug, episodic
30483	Combinations of drug dependence excluding opioid type drug, in remission
30490	Unspecified drug dependence, unspecified
30491	Unspecified drug dependence, continuous
30492	Unspecified drug dependence, episodic
30493	Unspecified drug dependence, in remission
30500	Alcohol abuse, unspecified
30501	Alcohol abuse, continuous
30502	Alcohol abuse, episodic
30503	Alcohol abuse, in remission

© 2024 Heissel JA et al. *JAMA Network Open.*

Excluded ICD9 Codes	Description of Excluded Codes
3051	Tobacco use disorder
30520	Cannabis abuse, unspecified
30521	Cannabis abuse, continuous
30522	Cannabis abuse, episodic
30523	Cannabis abuse, in remission
30530	Hallucinogen abuse, unspecified
30531	Hallucinogen abuse, continuous
30532	Hallucinogen abuse, episodic
30533	Hallucinogen abuse, in remission
30540	Sedative, hypnotic or anxiolytic abuse, unspecified
30541	Sedative, hypnotic or anxiolytic abuse, continuous
30542	Sedative, hypnotic or anxiolytic abuse, episodic
30543	Sedative, hypnotic or anxiolytic abuse, in remission
30550	Opioid abuse, unspecified
30551	Opioid abuse, continuous
30552	Opioid abuse, episodic
30553	Opioid abuse, in remission
30560	Cocaine abuse, unspecified
30561	Cocaine abuse, continuous
30562	Cocaine abuse, episodic
30563	Cocaine abuse, in remission
30570	Amphetamine or related acting sympathomimetic abuse, unspecified
30571	Amphetamine or related acting sympathomimetic abuse, continuous
30572	Amphetamine or related acting sympathomimetic abuse, episodic
30573	Amphetamine or related acting sympathomimetic abuse, in remission
30580	Antidepressant type abuse, unspecified
30581	Antidepressant type abuse, continuous
30582	Antidepressant type abuse, episodic
30583	Antidepressant type abuse, in remission
30590	Other, mixed, or unspecified drug abuse, unspecified
30591	Other, mixed, or unspecified drug abuse, continuous
30592	Other, mixed, or unspecified drug abuse, episodic
30593	Other, mixed, or unspecified drug abuse, in remission
3060	Musculoskeletal malfunction arising from mental factors
3061	Respiratory malfunction arising from mental factors
3062	Cardiovascular malfunction arising from mental factors
3063	Skin disorder arising from mental factors
3064	Gastrointestinal malfunction arising from mental factors
30650	Psychogenic genitourinary malfunction, unspecified
30651	Psychogenic vaginismus
30652	Psychogenic dysmenorrhea

© 2024 Heissel JA et al. *JAMA Network Open.*

Excluded ICD9 Codes	Description of Excluded Codes
30653	Psychogenic dysuria
30659	Other genitourinary malfunction arising from mental factors
3066	Endocrine disorder arising from mental factors
30720	Tic disorder, unspecified
30721	Transient tic disorder
30722	Chronic motor or vocal tic disorder
30723	Tourette's disorder
30781	Tension headache
3102	Postconcussion syndrome
3108	Nonpsychot brain syn, Not elsewhere classifiable
31081	Pseudobulbar affect
31089	Other specified nonpsychotic mental disorders following organic brain damage
31535	Childhood onset fluency disorder

Additional ICD9 Codes Used	Description of Added Codes
32700	Organic insomnia, Not otherwise specified
32701	Insomnia in other dis
32702	Insomnia dt mental disor
32709	Organic insomnia, Not elsewhere classifiable
32710	Organic hypersomnia, Not otherwise specified
32711	Idio hypersom-long sleep
32712	Idio hypersom-no lng slp
32713	Recurrent hypersomnia
32714	Hypersomnia in other dis
32715	Hypersom dt mental disor
32719	Organic hypersomnia, Not elsewhere classifiable
38845	Acq auditory process dis
7580	Down's syndrome
7581	Patau's syndrome
7582	Edwards' syndrome
75831	Cri-du-chat syndrome
75832	Velo-cardio-facial synd
75833	Microdeletions, Not elsewhere classifiable
75839	Autosomal deletions, Not elsewhere classifiable
75983	Fragile x syndrome
78002	Transient alteration of awareness
7801	Hallucinations
78050	Sleep disturbance, Not otherwise specified
78052	Insomnia, Not otherwise specified
78054	Hypersomnia, Not otherwise specified

© 2024 Heissel JA et al. *JAMA Network Open.*

Additional ICD9 Codes Used	Description of Added Codes
78055	Irreg sleep-wake rhy, Not otherwise specified
78056	Sleep stage dysfunctions
78058	Sleep rel move disor, Not otherwise specified
78059	Sleep disturbances, Not elsewhere classifiable
78460	Symbolic dysfunction, Not otherwise specified
78461	Alexia and dyslexia
78469	Symbolic dysfunction, Not elsewhere classifiable
797	Senility w/o psychosis
79921	Nervousness
79922	Irritability
79923	Impulsiveness
79924	Emotional lability
79925	Demoralization & apathy
79929	Emotional state sym, Not elsewhere classifiable
79951	Attn/concentrate deficit
79952	Cog communicate deficit
79954	Psychomotor deficit
79955	Frontal lobe deficit
79959	Cognition sign/sympt, Not elsewhere classifiable
V6284	Suicidal ideation
V7101	Obsv-adult antisoc behav
V7102	Obsv-adolesc antisoc beh
V7109	Observ-mental cond, Not elsewhere classifiable

© 2024 Heissel JA et al. *JAMA Network Open.*

eFigure 3. Comparing mothers and fathers to matches across the transition to parenthood, using primary diagnostic codes to define the outcome

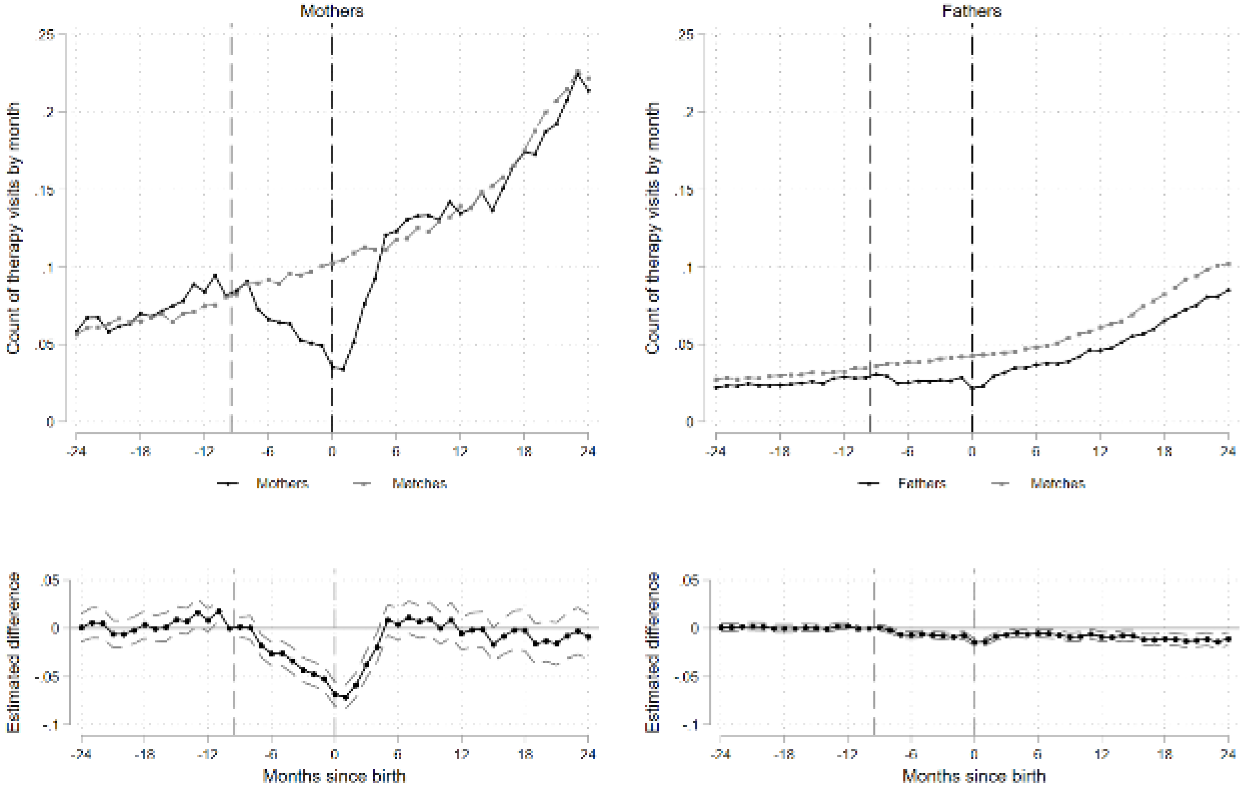

Notes: Panel A is mothers; Panel B is fathers. Top row displays the mean count of primary mental health care encounters for mothers and fathers based on diagnostic codes (black line) and their respective matches (gray line) by month relative to birth. Bottom row shows the regression-adjusted estimate of the difference between the parents and matches measured relative to 10 months before birth. Dashed gray line indicates 95% confidence interval in the bottom row. Data includes first births from January 1, 2014, to December 31, 2017; sample is required to be in the data from at least 12 months before to 24 months after birth.

© 2024 Heissel JA et al. *JAMA Network Open.*

eFigure 4. Comparing Army and Navy mothers to matches across the end of the 6- and 12-week leave policies, using primary diagnostic codes to define the outcome

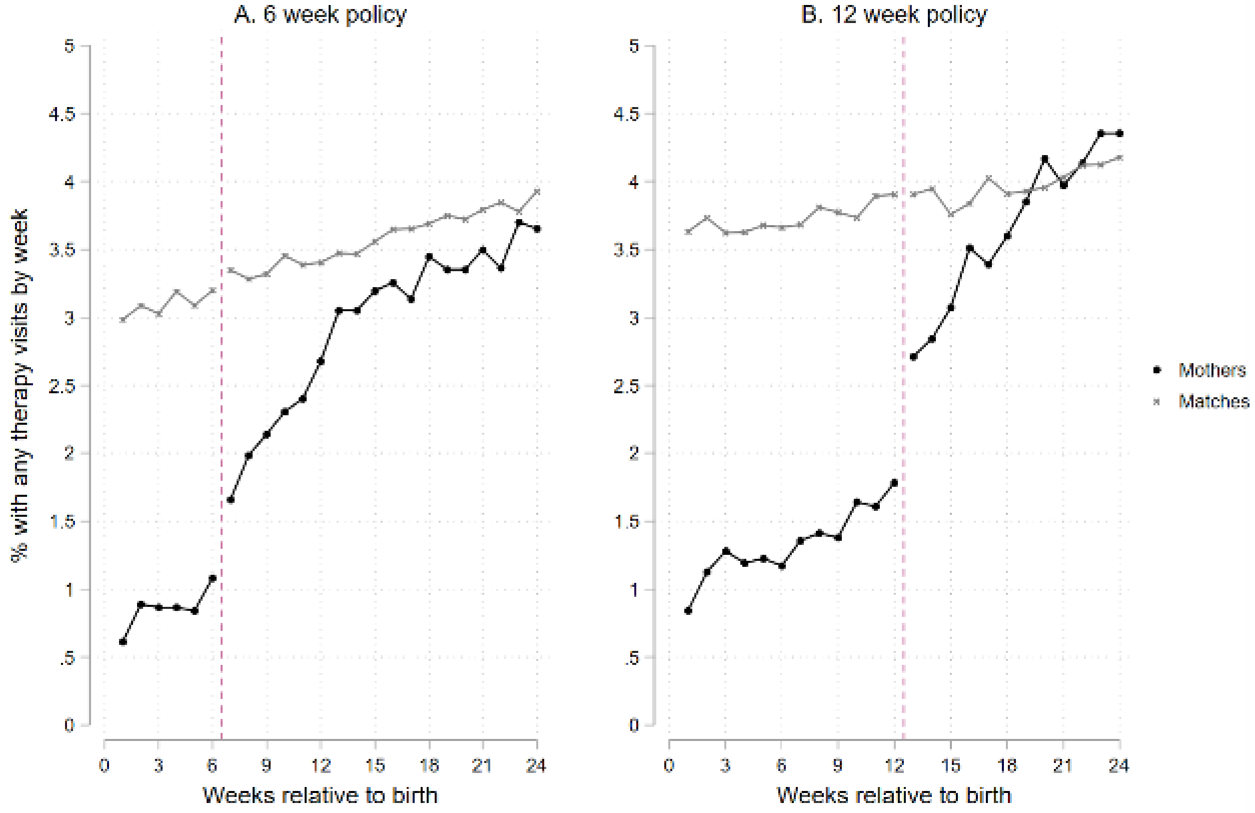

Notes: Displays the mean percent of mothers and their matches who had at least one primary mental health encounter (based on diagnostic codes) by week relative to birth. Panel A is the 6-week parental leave policy. Panel B is the 12-week parental leave policy. Day 1 of week 1 begins the first full day after discharge from the hospital to match the start of parental leave tracking for administrative purposes.

© 2024 Heissel JA et al. *JAMA Network Open.*
