# Supplementary material for: Mental Health Treatment Rates During Pregnancy and Post Partum in US Military Service Members
Source: JAMA Netw Open. 2024 May 30;7(5):e2413884. doi: 10.1001/jamanetworkopen.2024.13884 (PMC11140539; doi:10.1001/jamanetworkopen.2024.13884)
Supplement: Supplement 2. — Data Sharing Statement [file jamanetwopen-e2413884-s002.pdf]

Data Sharing Statement

Heissel. Mental Health Treatment Rates During Pregnancy and Post Partum in US Military Service Members. *JAMA Netw Open*. Published May 30, 2024. doi:10.1001/jamanetworkopen.2024.13884

Data
Data available: No

Additional Information
Explanation for why data not available: Data from the Department of Defense is not available for sharing with the public.
